# Supplementary material for: MetaRibo-Seq measures translation in microbiomes
Source: Nat Commun. 2020 Jun 29;11:3268. doi: 10.1038/s41467-020-17081-z (PMC7324362; doi:10.1038/s41467-020-17081-z)
Supplement: Supplementary file 10 — Supplementary Data 7 [file 41467_2020_17081_MOESM10_ESM.zip › File2/Confidence_High_Taxonomy/414645_out.krona.html]

Javascript must be enabled to view this page.

members
magnitude
magnitudeUnassigned
count
unassigned
taxon
rank

414645\_out

25

25
2
superkingdom

1224
25
phylum

1236
25
class

order
25
91347

family

SRS019129\_contig\_number\_6198SRS148159\_contig\_number\_1905
2
25
543

561
22

SRS098881\_contig\_number\_contig-100\_2798.159437
1
genus

562
21

SRS012273\_contig\_number\_37836SRS017191\_contig\_number\_13544SRS017521\_contig\_number\_43981SRS049402\_contig\_number\_15178SRS057478\_contig\_number\_1906SRS064276\_contig\_number\_26142SRS075947\_contig\_number\_11883SRS076876\_contig\_number\_108SRS077127\_contig\_number\_contig-100\_2862.95361SRS077454\_contig\_number\_8034SRS1041031\_contig\_number\_9883SRS140492\_contig\_number\_24818SRS142781\_contig\_number\_6380SRS143014\_contig\_number\_contig-100\_264.265SRS143596\_contig\_number\_contig-100\_287.288SRS144219\_contig\_number\_contig-100\_401.402SRS144362\_contig\_number\_24617SRS146888\_contig\_number\_15136SRS146996\_contig\_number\_10592SRS147022\_contig\_number\_14055SRS147151\_contig\_number\_5947
species

genus
1
620

species

SRS105082\_contig\_number\_2205
1
624
